# Profile of lysine acetylation in *Eimeria tenella* and its potential implications for anticoccidial research

**DOI:** 10.1186/s13071-026-07550-7

**Published:** 2026-07-07

**Authors:** Haibin Huang, Han Mo, Zhiyuan Zhang, You Yang, Wentao Yang, Yanlong Jiang, Nan Wang, Jianzhong Wang, Chunwei Shi, Xin Cao, Yan Zeng, Chunfeng Wang, Guilian Yang

**Affiliations:** 1 College of Veterinary Medicine, Jilin Agricultural University, Changchun, 130118 China; 2 Jilin Provincial Engineering Research Center of Animal Probiotics, Jilin Agricultural University, Changchun, 130118 China; 3 Jilin Provincial Key Laboratory of Animal Microecology and Healthy Breeding, Jilin Agricultural University, Changchun, 130118 China; 4 Engineering Research Center of Microecological Vaccines (Drugs) for Major Animal Diseases, Ministry of Education, Jilin Agricultural University, Changchun, 130118 China

**Keywords:** Anticoccidial effect, Acetylation, HDAC inhibitor

## Abstract

**Background:**

Coccidiosis has resulted in substantial economic losses in the poultry industry. The prevalence and severity of drug resistance to anticoccidial agents have exacerbated the challenges associated with controlling coccidiosis. Consequently, exploring novel drug targets is crucial for the prevention and treatment of coccidiosis.

**Methods:**

In this study, we performed acetylation modification proteomics on the sporozoites and merozoites of *Eimeria tenella* during the asexual stage. Moreover, the anticoccidial efficacy of histone deacetylase (HDAC) inhibitors was evaluated both in vitro and in vivo.

**Results:**

In total, 341 differentially acetylated proteins and 558 differentially acetylated modification sites were identified. Histones H2A, H2B, H2B variants, H3, and H4 are all acetylated and have multiple acetylation sites, suggesting that acetylation plays important roles in the gene regulation, replication, and development of *E. tenella*. KEGG pathway analysis revealed that glycolysis/gluconeogenesis pathway was enriched. Glycolysis is the main mode of energy metabolism in Apicomplexa. In the glycolysis pathway, eight key enzymes are downregulated by acetylation and have multiple acetylation sites. It has been suggested that histone deacetylase (HDAC) is the target of anticoccidial action. Trichostatin A (TSA, an inhibitor of HDAC family deacetylases) and nicotinamide (NAM, an inhibitor of SIRT family deacetylases) can inhibit sporozoite invasion (*P* ≤ 0.01) and promote sporozoite apoptosis (*P* ≤ 0.05). Intraperitoneal injection of TSA and NAM can reduce chicken caecal lesions and oocyst shedding, and the anticoccidial index can reach 143.

**Conclusions:**

This study is the first to investigate the anticoccidial effect of deacetylase inhibitors, providing a new strategy for the prevention and control of coccidiosis.

**Graphical Abstract:**

**Figure:**
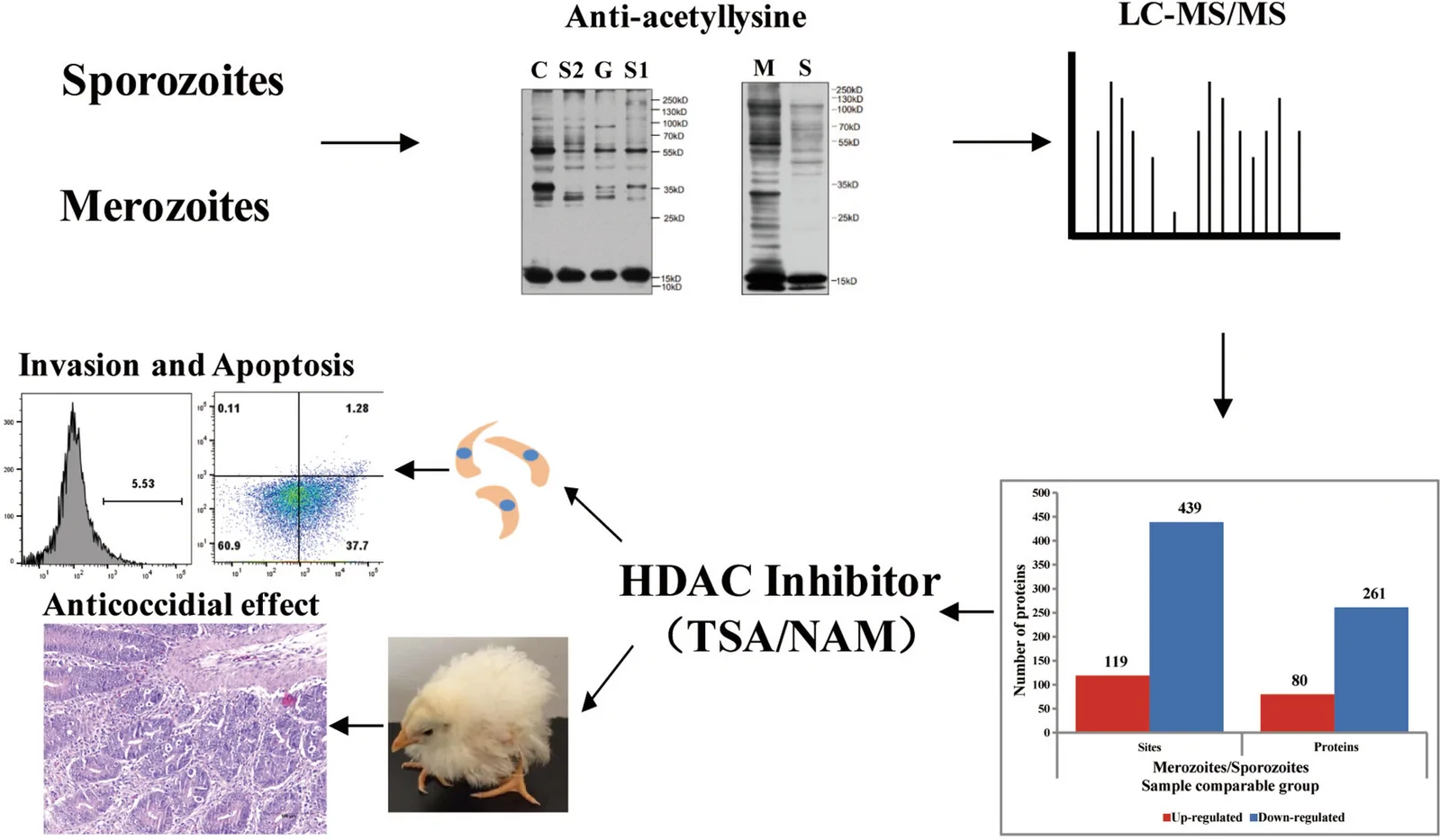

**Supplementary Information:**

The online version contains supplementary material available at https://doi.org/10.1186/s13071-026-07550-7.

## Background

Avian coccidiosis is a common intestinal disease in poultry caused by the genus *Eimeria* and causes substantial economic losses worldwide every year [1]. *E. tenella* is considered the most pathogenic of the seven *Eimeria* genera in chickens and causes severe tissue damage, bleeding, and diarrhea [2]. Coccidiosis can be prevented and treated with anticoccidial drugs and live oocyst vaccines consisting of nonattenuated and attenuated parasites [3]. However, these measures have drawbacks, including the emergence of drug resistance and the restoration of virulence from live vaccines, as well as their high cost [4]. These challenges have driven the development of new drug targets and control strategies. The life cycle of coccidia includes both asexual and sexual stages. During the asexual stage, merozoites proliferate massively and cause intestinal tissue damage. Therefore, screening drug targets at the asexual stage may be more effective.

Lysine acetylation is a reversible post-translational modification (PTM) that is involved in key cellular processes related to physiology and various diseases. It affects protein function through multiple mechanisms, including the regulation of protein stability, enzyme activity, cellular localization and crosstalk with other post-translational modifications [5]. Studies have shown that acetylation is a widespread PTM on the proteins of a variety of apicomplexan parasites, including *Toxoplasma gondii*, *Cryptosporidium,* and *Plasmodium*. Lysine acetylation modifies nearly 14% of *P. falciparum*, affecting proteins involved in multiple biological pathways [6].

Acetylation is coordinated by histone acetyltransferases (HATs) and histone deacetylases (HDACs) at the N-terminus of histone lysine residues [7]. HDACs are important in eukaryotic cells and play crucial roles in gene expression, transcription, and post-transcriptional protein modification by reducing histone acetylation levels [8]. These proteins include class I Rpd3-like proteins (HDAC1, HDAC2, HDAC3, and HDAC8), class II Hda1-like proteins (HDAC4, HDAC5, HDAC6, HDAC7, HDAC9, and HDAC10), class III Sir2-like proteins (SIRT1–7), and class IV proteins (HDAC11) [9]. Studies have shown that HDAC inhibitors are effective targets in a variety of protozoal diseases. The HDAC inhibitor MC1742 can inhibit the growth of *T. gondii* tachyzoites in vitro [10]. Moreover, the HDAC inhibitor Rac-AR-42 is a potent inhibitor of the growth of the asexual intraerythrocytic stage *P. falciparum* 3D7 and effectively inhibits the growth of *P. berghei*-infected in vitro erythrocytes and the extraerythrocytic form of *P. berghei* in hepatocytes. Oral administration of rac-AR-42 and the two achiral analogues inhibited *P. berghei* growth in mice, and rac-AR-42 (50 mg/kg/day as a single dose for 4 days) successfully cured all infections [11]. SAHA-OBn is a SAHA derivative of the O-alkyl hydroxamate family and is an inactive HDAC inhibitor of human HDAC subtypes. It was effective at reducing parasite loads in primary target tissues (bone marrow, spleen and liver) by more than 70% at 25 mg/kg via oral and intraperitoneal routes, and it was demonstrated to be more active than the reference compound (meglumine antimimonate, MA) and showed no toxicity [12].

In this study, TMT-based quantitative analysis of lysine acetylation was performed on sporozoites and merozoites of *Eimeria tenella*, and the differences in acetylation between histone and non-histone proteins were analyzed. Broad-spectrum deacetylase inhibitors, trichostatin A (TSA) and nicotinamide (NAM) [13], were utilized to investigate the biological role of lysine deacetylation in *E. tenella* and to evaluate the potential anticoccidial effects of the inhibitors. Our results provide new strategies for the study of coccidial and anticoccidial drug targets.

## Methods

### Parasite collection

Sixty 15-day-old AA chickens were obtained from the Dehui broiler chicken hatchery in Jilin Province. Chickens were raised in an environment at 20–26 °C with free access to food and water.

Ten chickens were orally infected with 5 × 10^4^ sporulated oocysts of *E. tenella* (Beijing strain) and sacrificed 120 h after infection. Each caecum was collected, rinsed with PBS to remove the caecal contents, digested with 1% hyaluronidase for 30 min, and filtered through gauze to remove large intestinal debris. The mixture was centrifuged at 3000 rpm for 10 min, the supernatant was discarded, and the pellet was resuspended in PBS. Purified merozoites were obtained by density gradient centrifugation as described in previous studies [14, 15]. Sporulated *E. tenella* oocysts were ground with glass beads to release the sporocysts. After centrifugation, the supernatant was discarded, and the pellet was resuspended in digestion solution and incubated at 41 °C for 20–40 min. Digestive fluid containing sporozoites was filtered through a G3 sand core funnel to collect the sporozoites [16]. The collected sporozoites and merozoites were snap-frozen in liquid nitrogen and stored at − 80 °C.

### Western blot analysis

Twenty chicks were used to obtain caecal samples at different time points post infection. Among them, fifteen chicks were orally inoculated with 5 × 10^4^ sporulated oocysts, and the remaining five chicks served as uninfected controls. The chicks were euthanized at 96, 120, and 144 h post infection, after which the caeca were collected. Caecum and *E. tenella* (sporozoite and merozoite) protein extracts were subjected to Western blotting as previously described [17]. The antibodies used in this study were as follows: anti-acetyllysine (PTM-101, Lot: 22838591HB14, 1:1000 dilution) and horseradish peroxidase-conjugated anti-mouse IgG (Thermo, 31430, 1:5000 dilution).

### Protein extraction and trypsin digestion

Sporozoites and merozoites were first pulverized in liquid nitrogen to obtain a cell powder, which was subsequently transferred to centrifuge tubes. To the powder, four volumes of lysis buffer (containing 8 M urea, 1% protease inhibitor cocktail, 3 μM TSA, 50 mM NAM, and 2 mM EDTA) were added. The mixture was kept on ice and subjected to three cycles of sonication using a high-intensity sonicator [18]. After centrifugation (12,000×*g*, 4 °C, 10 min), the supernatant was collected, and the protein concentration was measured using a BCA assay kit. The protein solution was then reduced with 5 mM dithiothreitol (DTT) at 56 °C for 30 min, followed by alkylation with 11 mM iodoacetamide in the dark at room temperature for 15 min. To dilute the protein sample below 2 M urea, 100 mM TEAB was added. Finally, the proteins were digested twice with trypsin [19].

### High-performance liquid chromatography (HPLC) fractionation and affinity enrichment

Tryptic peptides were fractionated into 6 fractions by high-pH reversed-phase HPLC using a Thermo Betasil C18 column (5 μm particles, 10 mm ID, 250 mm length), and the peptides were dried by vacuum centrifugation [20]. To enrich for modified peptides, fractionated peptides were added to NETN buffer (100 mM NaCl, 1 mM EDTA, 50 mM Tris–HCl, 0.5% NP-40, pH 8.0) and incubated with anti-acetyllysine antibody beads overnight at 4 °C with gentle shaking. The beads were then washed four times with NETN buffer and twice with H_2_O. Bound peptides from the beads were eluted with 0.1% trifluoroacetic acid, and the resulting peptides were desalted using C18 ZipTips (Millipore) after vacuum drying [21].

### LC‒MS/MS analysis

The tryptic peptides were reconstituted in 0.1% formic acid (solvent A) and then applied to a homemade reversed-phase analytical column (15 cm in length, 75 μm inner diameter). The mobile phase gradient was programmed as follows: solvent B (0.1% formic acid in 90% acetonitrile) was increased from 6 to 23% over 26 min, then from 23 to 35% over 8 min, followed by an increase to 80% in 3 min, and maintained at 80% for the final 3 min. This was carried out at a constant flow rate of 400 nL/min using an EASY-nLC 1000 UPLC system [22].

The peptides were analysed using a nanospray ionization (NSI) source coupled to tandem mass spectrometry (MS/MS) on a Q Exactive™ Plus (Thermo) instrument integrated online with the UPLC system. The electrospray voltage was set to 2.0 kV. Full scans were acquired within an m/z range of 350–1800, and intact peptides were detected in the Orbitrap at a resolution of 70,000. Selected peptides were further analysed by MS/MS with a normalized collision energy (NCE) of 28, and their fragments were identified in the Orbitrap at a resolution of 17,500. Data-dependent acquisition mode was employed, alternating between one full MS scan and 20 MS/MS scans, with a dynamic exclusion of 15.0 s. The automatic gain control (AGC) target was set to 5E4, and the fixed first mass was set to 100 m/z [23].

### Database search

The MS/MS data were analysed using the MaxQuant search engine (version 1.5.2.8). Tandem mass spectra were compared against the *E. tenella* UniProt database, which was combined with a reverse decoy database. Trypsin/P was chosen as the enzyme for peptide cleavage, allowing up to four missed cleavages. The precursor ion mass tolerance was set to 20 ppm for the first search and 5 ppm for the main search; the fragment ion mass tolerance was set to 0.02 Da. Carbamidomethylation of cysteine was designated as a fixed modification, and acetylation and oxidation of methionine were treated as variable modifications. The false discovery rate (FDR) was controlled to be less than 1%, and the minimum score for modified peptides was set to > 40. Proteins with quantitative ratios greater than 2 or less than 1/2 were considered significantly altered [24].

### Bioinformatics analysis

Gene Ontology (GO) annotations for the proteomes were obtained from the UniProt-GOA database (http://www.ebi.ac.uk/GOA/). Proteins identified in the analysis were annotated with GO terms using either the UniProt-GOA database or InterProScan software. These proteins were subsequently classified into three categories: biological processes, cellular components, and molecular functions [25].

The functional annotations of the identified protein domains were performed using InterProScan, a sequence analysis tool that employs protein sequence alignment techniques and the InterPro domain database (http://www.ebi.ac.uk/interpro/). In addition, protein pathways were annotated based on data from the Kyoto Encyclopedia of Genes and Genomes (KEGG) database [25]. We used WoLF PSORT, a subcellular localization prediction software, to predict subcellular localization [26].

Soft MoMo (motif-x algorithm) was used to analyse the sequence model composed of amino acids at specific positions to modify 21-mers (± 10 amino acid residues around the lysine acetylation site) in all protein sequences. All database protein sequences were used as background parameters to analyse changes in the frequency of amino acids near the modification sites.

Proteins were classified into three categories through GO annotation: biological processes, cellular compartments, and molecular functions. Enriched pathways were identified using a two-tailed Fisher's exact test with the KEGG database to assess the degree of enrichment of differentially modified proteins relative to all identified proteins. For each category of proteins, the InterPro database was consulted, and a two-tailed Fisher’s exact test was conducted to evaluate the enrichment of the differentially modified proteins among all identified proteins. A significance level of *P* < 0.05 indicated significant enrichment.

Protein functional clustering based on the differential modifications was analysed with reference to previous studies [23].

### Parasite invasion assay

DF-1 cells (1.5 × 10^6^ cells/well) were seeded into six-well plates and cultured at 37 °C in 5% carbon dioxide until the cell coverage reached 80%. The sporozoites were isolated according to method 2.1, incubated with carboxyfluorescein succinimide ester (CFSE, final concentration 10 μM) for 30 min at 37 °C, and then washed once with PBS. A control group, an *E. tenella* group (4.5 × 10^6^ sporozoites per well), and a TSA/NAM + *E. tenella* group (4.5 × 10^6^ sporozoites per well [27], TSA final concentration of 0.5 μM [28], NAM final concentration of 5 mM [29]) were established, with 4 repetitions in each group. The cell culture plate was incubated at 37 °C in 5% carbon dioxide for 12 h. The culture medium was discarded, the cells were washed three times with PBS and digested with 0.25% trypsin to obtain a single-cell suspension, and sporozoite invasion was detected by flow cytometry.

### Assessment of sporozoite apoptosis by flow cytometry

A total of 10^6^ sporozoites were incubated with TSA (0.5 μM) and NAM (5 mM) for 6 h, and a PE Annexin V Apoptosis Detection Kit was used to detect sporozoite apoptosis. In brief, the sporozoite suspension was centrifuged, and the supernatant was discarded. Then, 5 μl of PE-conjugated Annexin V and 5 μl of 7-AAD were added, the samples were incubated at room temperature for 15 min, 400 μl of binding buffer was added to each sample, and the samples were subjected to flow cytometry.

### In vivo evaluation of the anticoccidial effects of TSA and NAM

Thirty 15-day-old AA chickens were randomly divided into three groups (one cage per group), namely, the control, *E. tenella*, and TSA/NAM + *E. tenella* groups. Chickens in the *E. tenella* group were orally infected with sporulated oocysts of *E. tenella* (5.0 × 10^4^). Chickens in the TSA/NAM + *E. tenella* group were infected with sporulated oocysts of *E. tenella* (5.0 × 10^4^) and were injected intraperitoneally with TSA (500 μg/kg) and NAM (1500 mg/kg) once a day for 7 consecutive days [30, 31]. Chickens in the control group were orally administered an equal amount of PBS. On the 7th day after *E. tenella* infection, the chicks were sacrificed, and the anticoccidial effect of TSA/NAM was evaluated according to previous studies [32]. Briefly, the survival rate, relative weight gain rate, caecal lesion score, and number of oocysts per gram of faeces (OPG) were calculated. The anticoccidial index (ACI) was calculated as follows: (relative rate of weight gain + survival rate) − (lesion value + oocyst value).

Caecal samples from each group were collected and fixed in 10% buffered formalin for 24 h and then subjected to HE staining as previously described [33].

### Statistical analysis

All the data are expressed as the mean and standard deviation (mean ± SD). Statistical analysis was performed using one-way ANOVA with GraphPad Prism 5.0 (GraphPad Software). Differences were considered to be statistically significant when *P* < 0.05, highly significant when *P* < 0.01, and extremely significant when *P* < 0.001.

## Results

### Parasite abundance and host post-translational modifications in asexual stages of *E. tenella*

**Fig. 1:**
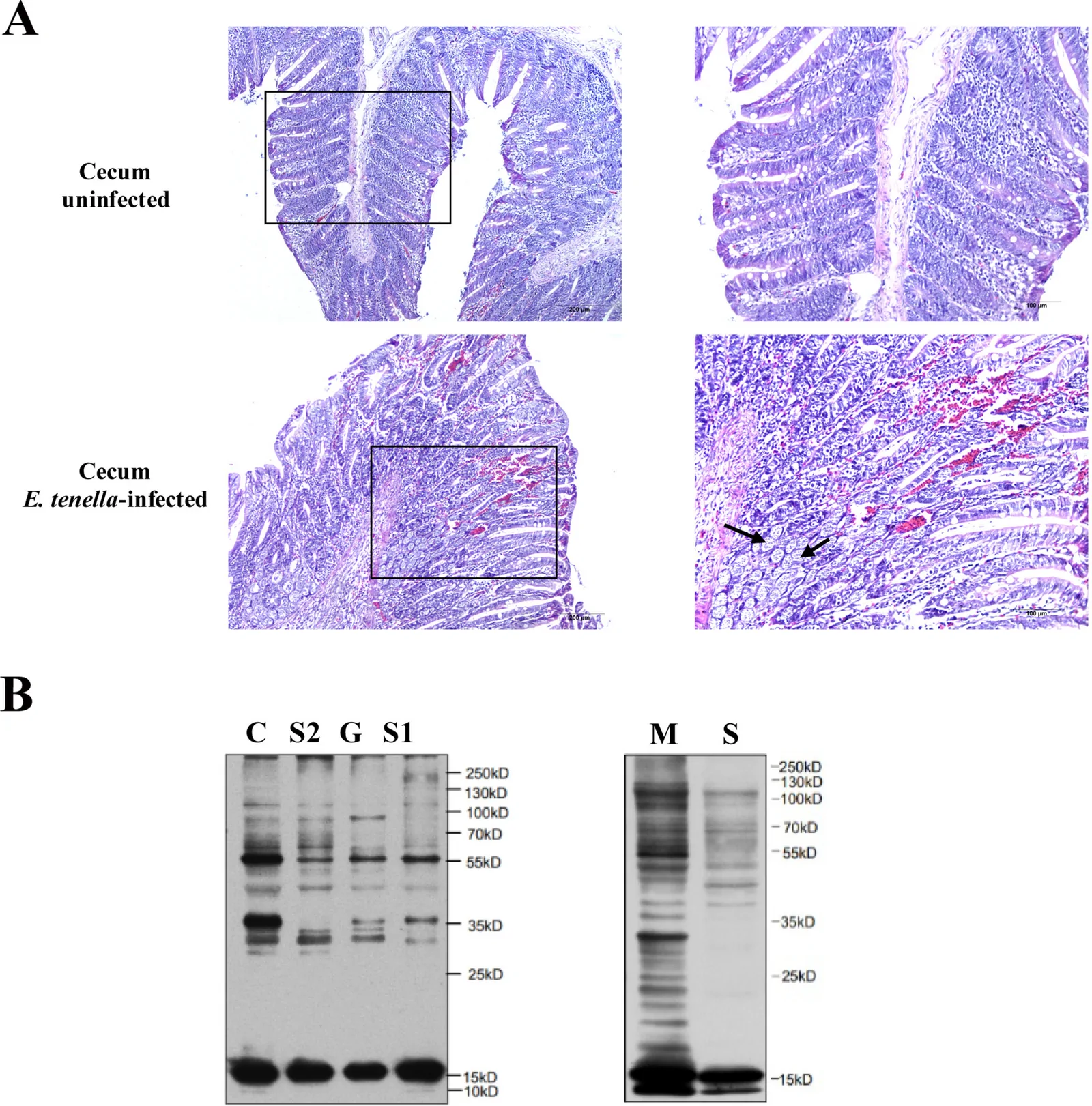
Intestinal pathology and lysine acetylation profiles during the asexual and sexual of *E. tenella.* **A** HE staining of chicken caecum 120 h after *E. tenella* infection. Black arrows: meronts. **B** Western blot analysis was performed to detect changes in acetylation levels in chicken cecum, sporozoites, and merozoites. C: chicken caecum of the control group. S2: Chicken caecum 120 h after infection with *E. tenella* (asexual stage: second-generation sporozoites). G: Chicken caecum 144 h after infection with *E. tenella* (sexual stage). S1: chicken caecum 96 h after infection with *E. tenella* (asexual stage: first-generation sporozoites). S: sporozoites. M: merozoites

After 120 h of infection with 5 × 10^4^ sporulated oocysts of *E. tenella*, massive haemorrhage of the caecum was observed, the epithelial mucosa was destroyed, and many red blood cells and schizonts were observed under a microscope (Fig. 1A).

Western blot analysis revealed the levels of acetylation in sporozoites, merozoites, and chicken caecal proteins (Fig. 1B). The results showed that, compared with untreated chicks, acetylation of host caecal proteins was altered during both the sexual and asexual stages of *E. tenella* infection. Significant changes in the acetylation of sporozoite and merozoite proteins were observed.

### Overview of the identification of acetylation modifications in sporozoites and merozoites of *E. tenella*

**Fig. 2:**
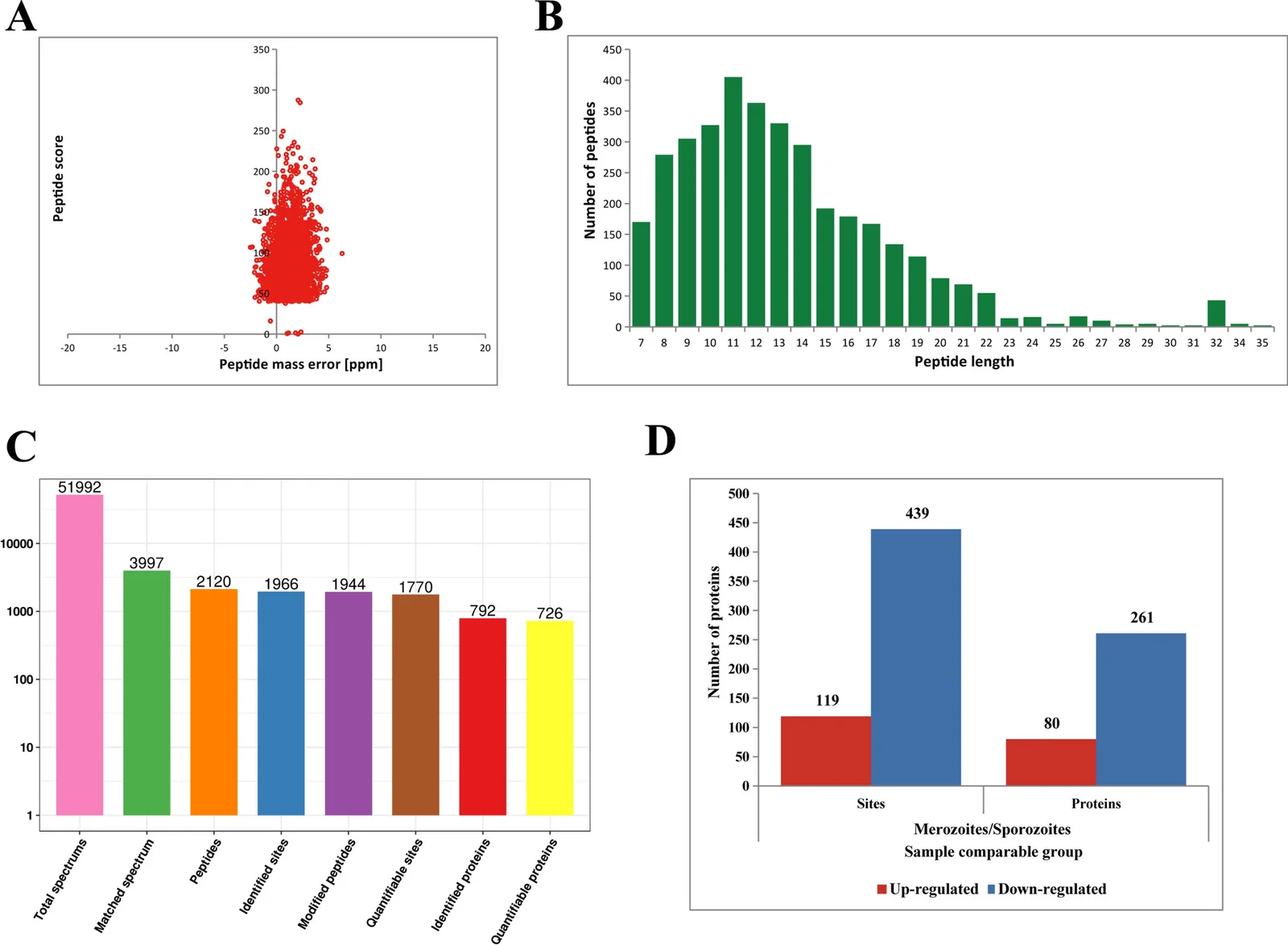
Overview of the identification of acetylation modifications in sporozoites and merozoites. **A** Mass spectrometry mass accuracy distribution. **B** Length distribution of peptides identified by mass spectrometry. **C** Basic statistical graph of the mass spectrometry data. **D** Histogram of the distribution of differentially modified proteins and modification sites in sporozoites and merozoites

Sporozoite and merozoite samples were subjected to protein extraction, trypsin digestion, TMT labelling, HPLC classification, modification enrichment, LC–MS/MS analysis, and bioinformatics analysis. The primary mass error of most spectra was within 10 ppm, indicating that the mass accuracy of the mass spectrometer was normal and that the qualitative and quantitative analysis of proteins was accurate (Fig. 2A). Most of the lysine-acetylated peptides identified by mass spectrometry were between 7 and 20 amino acids long (Fig. 2B). The distribution of peptide fragments conforms to the general rules, and the distribution of peptide fragment lengths meets the quality control requirements. A total of 1966 acetylation sites on 792 proteins were identified by mass spectrometry, of which 1770 sites on 726 proteins had quantitative information (Fig. 2C). A change in differential expression exceeding 2 was regarded as the change threshold for significant upregulation, and a change of less than 1/2 was regarded as the change threshold for significant downregulation. A total of 341 differentially acetylated proteins were identified, of which 80 were upregulated and 261 were downregulated, and 558 differentially acetylated modification sites were identified, of which 119 were upregulated and 439 were downregulated (Fig. 2D). The high number of acetylated proteins and sites in sporozoites and merozoites indicates that acetylation is important during the asexual stage of *E. tenella*.

### Subcellular localization and functional annotation of differentially acetylated proteins in sporozoites and merozoites

**Fig. 3:**
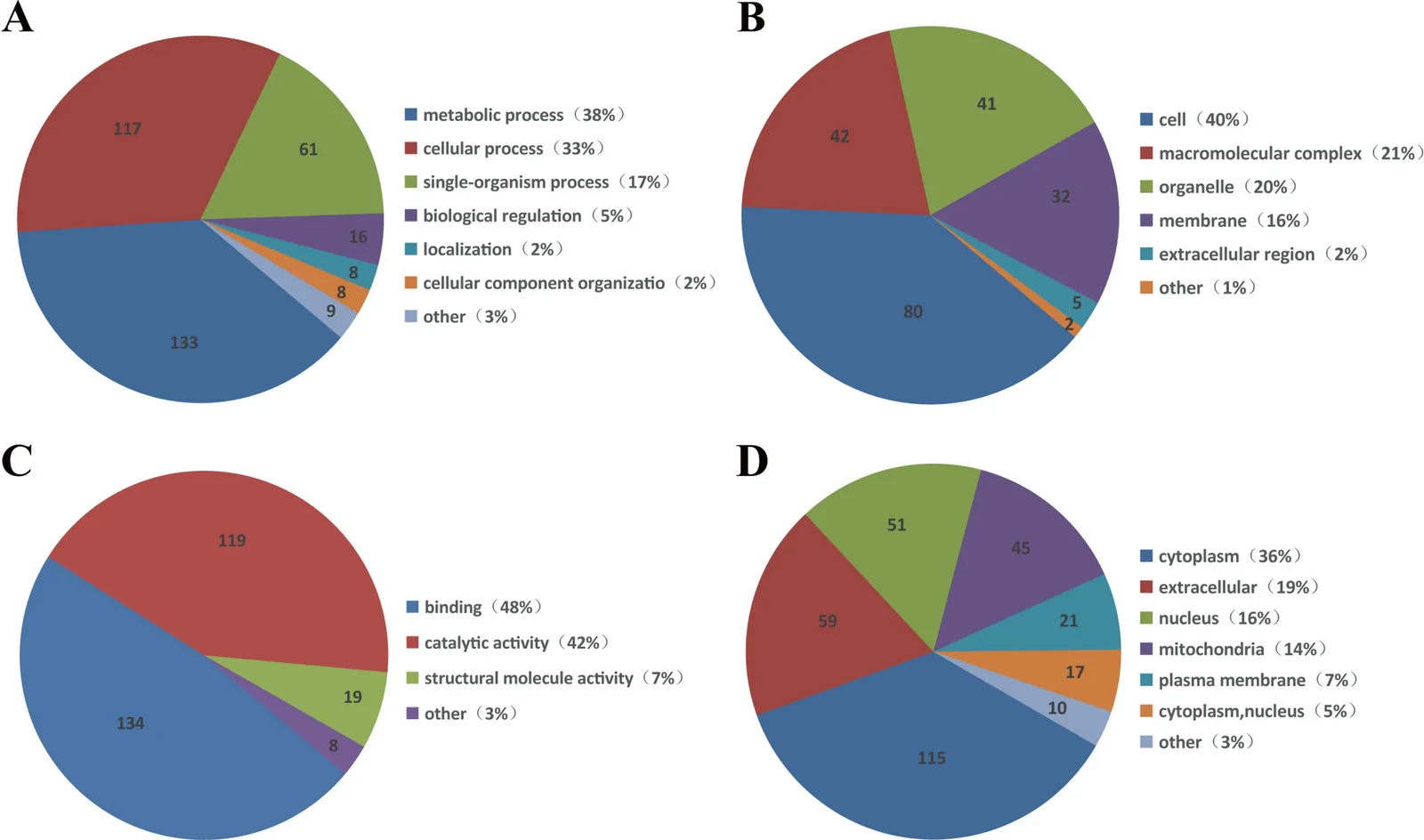
GO analysis and subcellular localization of differentially acetylated proteins in sporozoites and merozoites. **A** Biological process. **B** Cellular component. **C** Molecular function. **D** Subcellular localization

The results of the GO functional classification of the acetylated proteins revealed that the differentially acetylated proteins of *E. tenella* sporozoites and merozoites were involved in metabolic processes (38%), cellular processes (33%), single-organism processes (17%) (Fig. 3A). Differentially acetylated proteins were distributed in cellular components, mainly in cells (40%), macromolecular complexes (21%), organelles (20%), membranes (16%), and extracellular regions (2%) (Fig. 3B). The molecular functions of these acetylated proteins included mainly binding (48%), catalytic activity (42%), and structural molecule activity (7%) (Fig. 3C).

To understand the distribution of acetylated proteins in the subcellular structure of the parasite, we performed a statistical analysis of the subcellular structural positions of the differentially acetylated proteins in *E. tenella* sporozoites and merozoites. The results revealed that the differentially acetylated proteins were located mainly in the cytoplasm (36%), extracellular space (19%), nucleus (16%), mitochondria (14%), plasma membrane (7%), cytoplasm, nucleus (5%), and peroxisome (3%) (Fig. 3D).

### Functional enrichment analysis of differentially acetylated proteins in *E. tenella* sporozoites and merozoites

**Fig. 4:**
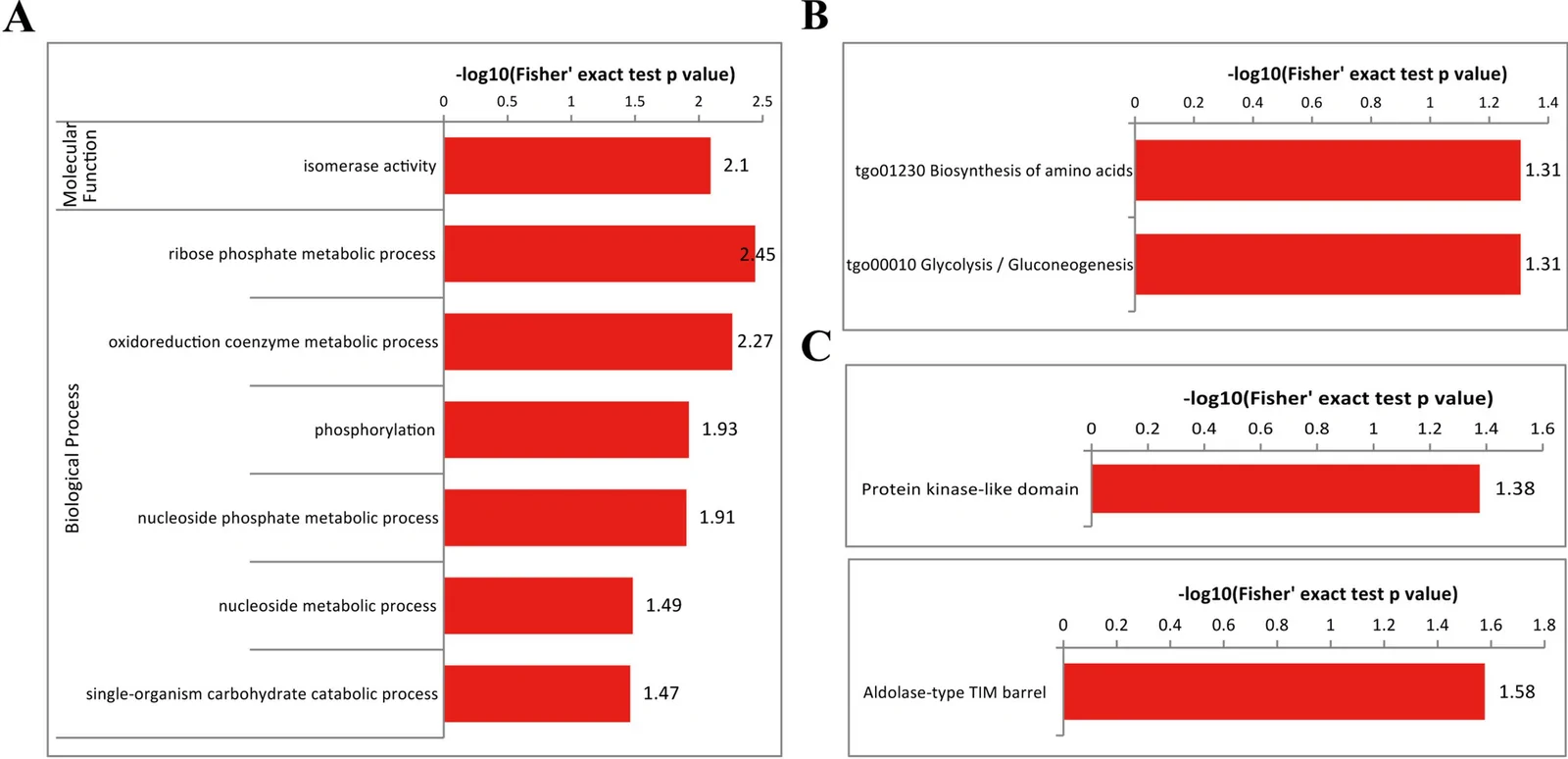
Enrichment analysis of differentially acetylated proteins in sporozoites and merozoites. **A** GO-based enrichment of differentially acetylated proteins in sporozoites and merozoites in terms of molecular functions, cellular components, and biological processes. **B** KEGG pathway enrichment analysis of differentially acetylated proteins in sporozoites and merozoites. **C** Protein domain enrichment analysis of differentially acetylated proteins in sporozoites and merozoites

GO enrichment analysis revealed that the differentially acetylated proteins in *E. tenella* sporozoites and merozoites were enriched mainly in ribose phosphate metabolic process, oxidoreduction coenzyme, metabolic process, phosphorylation, nucleoside phosphate metabolic process, nucleoside metabolic process, and single-organism carbohydrate catabolic process. Molecular functions were enriched in the sporozoites and merozoites differentially acetylated proteins, which mainly included isomerase activity (Fig. 4A).

To understand the functions of the differentially acetylated proteins, we performed KEGG pathway enrichment analysis. Differentially acetylated proteins were involved mainly in the biosynthesis of amino acids and in glycolysis/gluconeogenesis (Fig. 4B). Differentially acetylated proteins in the amino acid biosynthesis pathway were concentrated in enzymes related to glycolysis, gluconeogenesis, and the tricarboxylic acid cycle, such as pyruvate kinase, aconitate hydratase, isocitrate dehydrogenase, and transketolase. The acetylation of most sites in these proteins was downregulated; only the acetylation of aspartate aminotransferase and isocitrate dehydrogenase modification sites was upregulated (Table S1).

Domain enrichment analysis revealed that the upregulated acetylated protein domain was a protein kinase-like domain and that the downregulated acetylated protein domain was enriched in the aldolase-type TIM barrel (Fig. 4C). These findings indicate that the functions of these domains are regulated by acetylation. Acetylation of the membrane attack complex/perforin (MACPF) domain, the Rhoptry kinase family protein ROP25, and the Rhoptry kinase family protein ROP17 in the protein kinase-like domain was upregulated, indicating that acetylation may be related to the coccidial invasion of cells (Table S2).

### Lysine acetylation analysis of sporozoite and merozoite histones

Proteomic analysis revealed that the levels of histones H3, H2B, H2A, H2A variants, and H4 significantly differed between *E. tenella* merozoites and sporozoites, indicating that histone changes during the asexual stage of *E. tenella* play important roles in coccidial development. An acetylation modification omics study revealed that five histones of sporozoites and merozoites were acetylated and had multiple modification sites (Table 1). Acetylation modifications were observed on lysine residues of histone H3 at positions 113, 27, 58, 25, 10, 19, 82, and 15. Histone H2B was found to undergo acetylation at lysine residues 112, 71, 108, and 100. Acetylation modifications were detected at lysine residues 55, 19, 9, 115, 15, and 14 of the histone variant H2B. Histone H2A exhibited acetylation modifications at lysine residues located at positions 27, 18, 34, 24, 10, 29, and 14. Acetylation modifications were observed on lysine residues 6, 33, 13, 9, and 17 of histone H4. Sequence analysis of the modification sites revealed that alanine, glycine, and serine were predominantly located around the histone H3 lysine acetylation sites. Many aliphatic amino acids (alanine, glycine, and valine) are present around the lysine acetylation modification sites of histones H2B, H2A, and H4. The results of histone acetylation modification indicate that acetylation plays an important role in gene regulation in the asexual stage of *E. tenella*.

**Table 1:** Differential acetylated histones, modification sites and modified sequences in sporozoites and merozoites

Protein description	Gene name	Regulated type	Position
Enolase	ENO	Down	406, 342, 264, 246, 206, 131
Pyruvate kinase	ETH_00011145	Down	310, 211, 210, 123, 102, 59
Phosphoglycerate mutase	ETH_00039380	Down	227, 135, 61, 18
diphosphate-dependent phosphofructokinase	ETH_00003915	Down	667, 428, 235, 195
Triosephosphate isomerase	ETH_00011150	Down	239, 70
Fructose-bisphosphate aldolase	ETH_00008600	Down	38, 24
Phosphoglycerate kinase	ETH_00027415	Down	158
Glyceraldehyde-3-phosphate dehydrogenase	ETH_00011158	Down	229

### Acetylation modification of *E. tenella* sporozoite and merozoite proteins in the glycolysis/gluconeogenesis pathway

**Fig. 5:**
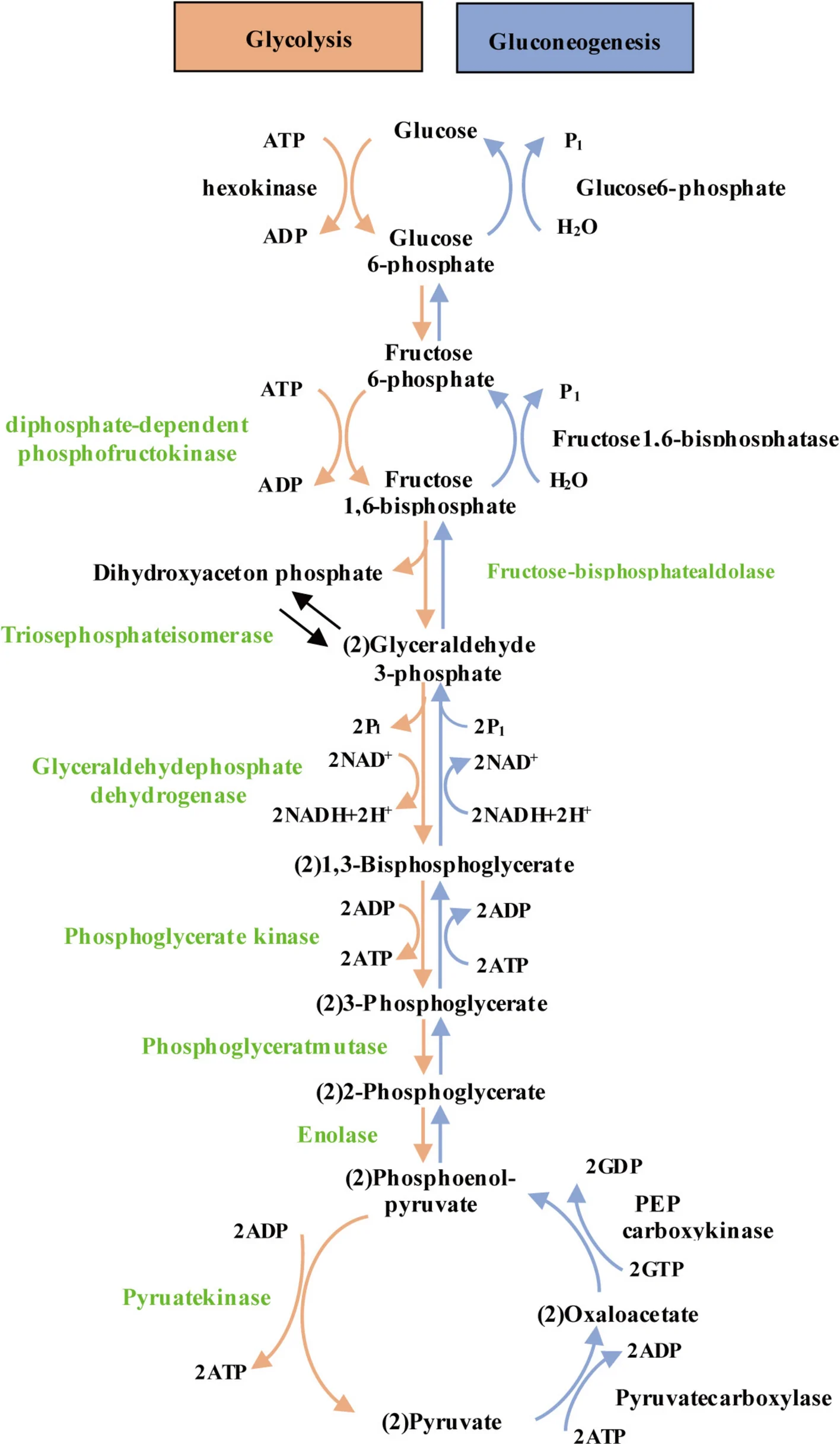
Acetylation modifications of key enzymes in the glycolytic pathway of sporozoites and merozoites. Green indicates enzymes with downregulated acetylation

Glycolysis is the main mechanism through which parasites obtain ATP. KEGG pathway enrichment analysis revealed that our results were enriched in the glycolysis/gluconeogenesis pathway, with eight differentially acetylated proteins in the glycolysis pathway between sporozoites and merozoites. These key enzymes regulate glycolysis, including enolase, pyruvate kinase (PK), phosphoglycerate mutase (PGAM), diphosphate-dependent phosphofructokinase (NTP-PFK), triosephosphate isomerase (TIM), fructose-bisphosphate aldolase (ALDO), phosphoglucomutase (PGM), phosphoglycerate kinase (PGK), and glyceraldehyde-3-phosphate dehydrogenase (GAPDH) (Fig. 5). These findings indicate that the asexual stage of *E. tenella* may regulate energy metabolism by regulating the acetylation of key enzymes involved in glycolysis. Enolase and PK have six acetylation modification sites, whereas PGAM and NTP-PFK have four acetylation modification sites. All acetylation modification sites of these enzymes were downregulated, indicating that histone deacetylases play important roles in energy metabolism in merozoites (Table 2).

**Table 2:** Acetylation modifications of key enzymes of the sporozoite and merozoite glycolytic pathways

Protein description	Gene name	Position	Modified sequence
Histone H2B	ETH_00028290	112, 71, 108, 100	AVTK(1)YTGK, IASEAGK(1)LCK, HAVSEGTK(1)AVTK, LVLPGELAK(1)HAVSEGTK
Histone H2B variant 1	ETH_00030960	53, 19, 9, 115, 15, 14	QVHPETGVSK(1)K, TAGK(1)SLGPR, GPAQK(1)SQAAK(1)K, LLLPGELSK(1)HAVSEGTK, SQAAK(1)K(1)TAGK, SQAAK(1)K(1)TAGK
Histone H3	ETH_00016640	123, 28, 57, 24, 80, 19, 10, 15	VTIMPK(1)DIQLAR, K(1)SAPVAGGIK, YQK(1)STDLLIR, K(1)QLASK(1)AAR, K(1)STGGK(1)APR, K(1)QLASK(1)AAR, EIAQDFK(1)TDLR, STGGK(1)APR
Histone H4	ETH_00028080	6, 32, 9, 13, 17	GK(1)GGK(1)GLGK, DNIQGITK(1)PAIR, GLGK(1)GGAK(1)R, GK(1)GGK(1)GLGK, GLGK(1)GGAK(1)R

### Anticoccidial effects of TSA and NAM in vivo and in vitro

**Fig. 6:**
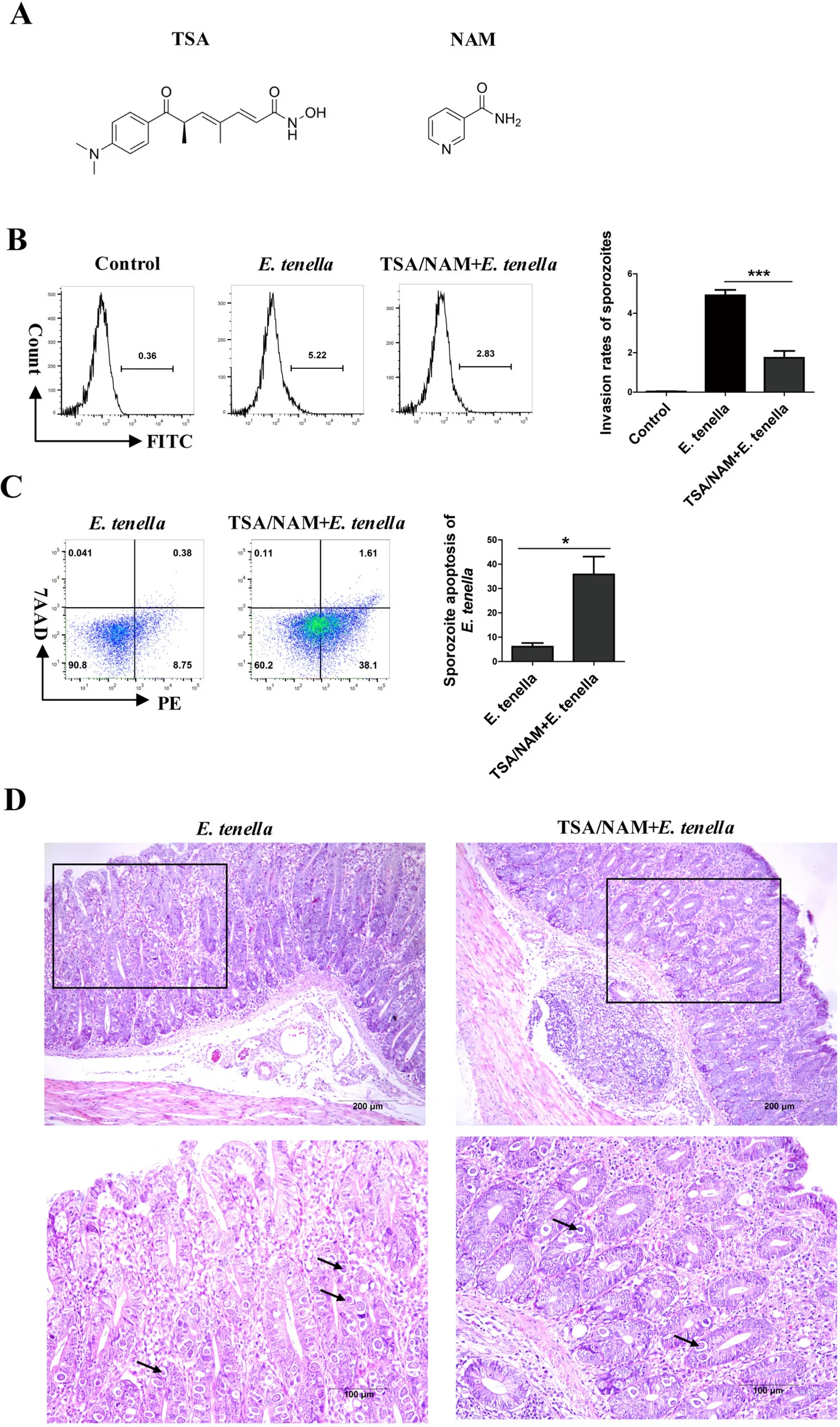
Anticoccidial effects of TSA and NAM in vivo and in vitro. **A** Structure of the HDAC inhibitor used in this study. **B** Flow cytometry was used to detect the effects of TSA and NAM on sporozoite invasion. **C** Effects of TSA and NAM on sporozoite apoptosis. **D** H&E staining of chicken caecum on Day 7 after *E. tenella* infection (representative of 2 experiments; *n* = 10/group). Black arrows: oocysts. Statistical significance was determined by one-way ANOVA. *P < 0.05; **P < 0.01; ***P < 0.001

Multiple key pathways, including those involving glycolysis and invasion-associated virulence factors, exhibited pronounced downregulation of acetylation. To preliminarily investigate the effects of deacetylases on the parasite, broad-spectrum deacetylase inhibitors (TSAs/NAMs) were selected for in vivo and in vitro experiments. The anticoccidial effects of the deacetylase inhibitors trichostatin A (TSA) and niacinamide (NAM) were investigated (Fig. 6A). The results of the sporozoite cell invasion experiment revealed that TSA and NAM significantly reduced the sporozoite invasion rate (*P* ≤ 0.01) (Fig. 6B). When TSA and NAM were incubated with sporozoites for 6 h, early apoptosis occurred in the sporozoites (*P* ≤ 0.05) (Fig. 6C), indicating that TSA and NAM inhibited coccidial invasion and proliferation.

Chickens infected with *E. tenella* were treated with either TSA or NAM. The results revealed that the weight of chicks in the TSA/NAM group was greater than that in the *E. tenella* group; however, the difference was not significant. The caecal villi of the chickens in the *E. tenella* group were shortened, and many oocysts were present. The intestinal pathology in the TSA/NAM group was significantly improved, and fewer oocysts were detected (Fig. 6D). The oocyst excretion of chicks in the TSA/NAM group was significantly reduced, intestinal lesions were alleviated, and the anticoccidial index was 143, indicating a moderate anticoccidial effect (Table 3).

**Table 3:** Lesion score (LS), relative body weight gain, number of oocysts per gram of faeces (OPG) and anticoccidial index (ACI) of chickens in each group

Group	Survival rates (%)	Relative weight gain	OPG (10^6^)	LS of cecum	ACI
Control	100	440.7 ± 50.67^a^	0	0	200
*E. tenella*	100	199.3 ± 24.32^b^	2.98 ± 0.23^A^	2.5 ± 0.22^A^	80
TSA/NAM + *E.tenella*	100	289.7 ± 25.67^b^	1.01 ± 0.27^B^	1.3 ± 0.21^B^	143

A, B: significantly different, *P* < 0.01; a, b: significantly different, *P* < 0.05. The presence of the same character indicates that there was no significant difference between the groups. (representative of 2 experiments, *n* = 5/group)

## Discussion

The caecum is specifically targeted by *E. tenella*, where sporozoites break through the physical barrier of the intestine, invade epithelial cells, and destroy intercellular barriers [34, 35]. The schizogony stage, which begins next, involves two to three rounds of asexual replication, resulting in the production of multinucleated schizonts [36]. Host cells containing second-generation schizonts separate from adjacent cells and migrate deep into the lamina propria to develop [37]. These processes often result in extensive bleeding of the intestines and massive destruction of intestinal cells [38]. This study focussed on the acetylation modification of the asexual stage (schizogony) of *E. tenella*, analysed the differences in the acetylation modification of sporozoite and merozoite proteins, and used the histone deacetylase inhibitors TSA and NAM to conduct in vivo and in vitro studies, confirming its anticoccidial effect.

Apicomplexan parasites, such as *Toxoplasma gondii* and *E. tenella*, undergo complex life cycles involving multiple stages, morphologies, and host cells. One of the mechanisms through which these parasites adapt to different host environments, evade host immune responses, and rapidly transition through their life cycles involves PTMs [39]. In the field of epigenetics, the most prominent form of chromatin modification is the covalent modification of histones, which includes phosphorylation, methylation, acetylation, and ubiquitination [40]. Among these modifications, histone acetylation has a profound effect on the expression of parasite genes. Highly conserved core histones (H2A, H2B, H3, and H4) and histone variants play crucial roles in DNA repair, transcription regulation, and parasite development [41]. For instance, the H2AX variant of *T. gondii* histones has been shown to induce the production of bradyzoites, a dormant form of the parasite, in vitro [42]. Histones H3 and H4, which are highly conserved, also play important roles in the cell cycle, gene expression, and parasite development [43, 44]. In *T. gondii*, histones H3 and H4 undergo acetylation modifications at multiple lysine residues, with acetylation occurring at lysine residues 9, 14, and 18 of H3 and at lysine residues 8 and 12 of H4 [45, 46]. Similarly, we observed that histones H2A, H2B, H3, and H4 of *E. tenella* schizonts (the asexual stage of the parasite) underwent acetylation modifications and had multiple acetylation sites. Importantly, when compared with *T. gondii*, *E. tenella* schizonts presented more acetylation sites, suggesting that acetylation modification plays crucial roles in gene regulation, replication, and development during schizogony.

Our findings on the acetylation of sporozoite and merozoite histones differed significantly from those of previous research. The differentially acetylated histones in previous studies were H2A, H2B, and H4, and this differentially acetylated in this study were H2A, H3, and H4. Furthermore, we observed notable differences in the acetylation sites. Previous studies have identified eight (K34, K6, K10, K14, K27, K29, K18 and K24), five (K9, K14, K15, K19 and K4), and four (K9, K13, K17 and K6) differential acetylation sites for H2A, H2B, and H4, respectively [23]. However, our study identified 1 (K34), 4 (K28, K57, K10, and K15), and 5 (K6, K32, K9, K13, and K17) acetylation sites for H2A, H3, and H4, respectively. Interestingly, we found that the K34 site of H2A was downregulated, which contrasts with the findings of previous research [23]. These differences may be attributed to factors such as the tender strain of *E. tenella* used and chicken age and breed. Further research is necessary to determine the reasons for these variations.

Energy metabolism has always been an important target for research on drugs against parasites, such as *Plasmodium*, filarial worms, *Trypanosoma cruzi*, and *Cryptosporidium parvum* [47–50]. Glycolysis, a process catalysed by ten enzymes, drives the conversion of glucose into pyruvate and leads to the production of the high-energy molecules adenosine monophosphate (ATP) and NADH [51]. In our study, the acetylation of eight enzymes (steps 3–10 of glycolysis) in glycolysis during the asexual stage of *E. tenella* was downregulated. PGK and PK convert 1,3-bisphosphoglycerate into 3-phosphoglycerate and phosphoenolpyruvate into pyruvate, respectively, which are the two ATP-generating steps in glycolysis [52]. PK is the rate-limiting step in glycolysis catalysis. PK inhibitors reduce the parasite load and ameliorate intestinal pathology in immunocompromised mice infected with *C. parvum* [53]. Studies have shown that *Babesia microti* pyruvate kinase I (BmPKI) activity is inhibited by tannic acid, apigenin, shikonin, and PKM2 inhibitors, which can reduce the growth of *B. microti* in vitro [1]. Enolase catalyses the dehydration of 2-phosphoglycerate (2-PGA) to phosphoenolpyruvate (PEP) [54]. Enolase antibodies inhibit the growth of *Plasmodium yoelii* and *Plasmodium berghei* and reduce parasitaemia in mice [55].

Studies have shown that multiple enolase inhibitors are trypanocides [56]. Small-molecule allosteric inhibitors of trypanosome phosphofructokinase (PFK) block the glycolytic pathway to achieve fast parasite kill times, and a single daily oral dose can cure parasitaemia in stage 1 mouse models of human African trypanosomiasis [57]. The phosphofructokinase activities of *Toxoplasma gondii*, *E. tenella*, and *Cryptosporidium parvum* are PPi dependent [58], and *T. gondii* PPi-PFK is critical for endoparasite reproduction and virulence [59]. A TIM inhibitor screened through molecular dynamics simulation and molecular docking analysis showed trichomonacidal and antitrypanosome effects [60, 61]. The mRNA expression of fructose-bisphosphate aldolase (FBA) was highest in second-generation merozoites of *E. tenella*, whereas the protein level was highest in unsporulated oocysts and was distributed mainly in the sporophyte cytoplasm. Morin is an FBA inhibitor that can significantly improve caecal lesions and reduce oocyst excretion in chickens infected with *E. tenella*, with an anticoccidial index of 162 [62]. GAPDH is distributed on the surface of sporozoites and merozoites as well as in the cytoplasm of merozoites and is a good protective antigen for *Eimeria* vaccines [63, 64]. Many studies targeting key enzymes of glycolysis as drug and vaccine targets have been reported in protozoa. However, the effects of acetylation modifications of these key enzymes in protozoa, especially the effects of histone acetyltransferases and histone deacetylases on Apicomplexa glycolysis, are less well known. Studies have shown that parasite glycolytic enzymes are involved in various important parasite–host interactions, such as parasite adhesion and invasion [65]. Glycolytic enzymes of *Plasmodium berghei* and *Plasmodium falciparum* recognize and bind to epithelial receptors, and anti-ENO antibodies inhibit parasite invasion [66]. This study revealed that TSA and NAM could inhibit *E. tenella* sporozoite invasion and promote sporozoite apoptosis, indicating that deacetylase inhibitors may inhibit glycolytic enzyme function and affect parasite invasion and survival.

Acetylation is a reversible process regulated by histone acetyltransferases (HATs) and histone deacetylases (HDACs) that control the levels of histone acetylation. Inhibitors of histone-modifying enzymes can specifically impede post-translational modifications, affecting gene transcription and development in parasitic organisms. Studies have shown that multiple HDAC inhibitors possess antiparasitic activities. Treatment of *T. cruzi* with FR235222, an HDAC inhibitor, inhibits parasite growth and differentiation [67]. Similarly, treatment of *C. parvum* with varinostat resulted in 92.3% irreversible inhibition of parasite growth [68]. Trichostatin A (TSA), another HDAC inhibitor, effectively inhibited gametocyte maturation in *Plasmodium falciparum*, upregulated the expression of genes associated with invasion, cell adhesion, and protein export, and downregulated the expression of transcription- and translation-related genes [69]. H4K8ac is a major regulator of chromatin-associated transcriptional changes during the intraerythrocytic developmental cycle (IDC) of *P. falciparum* and is associated with both constitutive chromatin and heterochromatic environments. PfH4K8ac is highly sensitive to TSA, which can alter the transcription of H4K8ac, with differentially expressed genes that are significantly enriched within ORFs, including genes associated with DNA replication, excision repair, and ribosome structure [70]. Our study found that both TSA and nicotinamide (NAM) can reduce intestinal damage and oocyst excretion in chickens.

## Conclusions

In this study, TSA/NAM significantly reduced the intestinal lesion scores and oocyst output in chicks; however, because the weight gain rate did not significantly improve, TSA/NAM had only a moderate anticoccidial effect. Previous studies have shown that acetylation influences animal body weight [71]. TSA and NAM can broadly inhibit histone deacetylase activity, which may impair host histone deacetylases and thereby affect chick growth and development. Therefore, systematic analysis of the acetylation sites of histones and key glycolytic enzymes during the asexual stage of *E. tenella*, together with the identification of histone deacetylase subtypes, is highly important for elucidating the anticoccidial mechanism of HDAC inhibitors. At present, *Eimeria* lacks an in vitro model capable of completing its full life cycle, which makes it difficult to validate modification sites and histone deacetylase subtypes and limits the screening of histone deacetylase inhibitors with anticoccidial activity.

## Supplementary Information

**Additional file 1: Table S1.** KEGG pathway enrichment analysis of differentially acetylated proteins in sporozoites and merozoites. **Table S2.** Protein domain enrichment analysis of differentially acetylated proteins in sporozoites and merozoites.

## Data Availability

Acetylation modification omics data were uploaded to the China National Center for Bioinformatics (https://ngdc.cncb.ac.cn/omix/submitList, OMIX ID: OMIX007908).
