# Supplementary material for: Profile of lysine acetylation in Eimeria tenella and its potential implications for anticoccidial research
Source: Parasit Vectors. 2026 Jul 7;19:419. doi: 10.1186/s13071-026-07550-7 (PMC13625346; doi:10.1186/s13071-026-07550-7)
Supplement: Supplementary file 1 [file 13071_2026_7550_MOESM1_ESM.doc]

Table S1 KEGG pathway enrichment analysis of differentially acetylated proteins in sporozoites and merozoites.

| **Domain Description** | **Protein description** | **Protein accession** | **Regulated Type** | **Position** |
| --- | --- | --- | --- | --- |
| tgo01230 Biosynthesis of amino acids | Phosphoglycerate mutase | U6L3D6 | Down | 18 |
| tgo01230 Biosynthesis of amino acids | Phosphoglycerate mutase | U6L3D6 | Down | 61 |
| tgo01230 Biosynthesis of amino acids | Phosphoglycerate mutase | U6L3D6 | Down | 227 |
| tgo01230 Biosynthesis of amino acids | Phosphoglycerate mutase | U6L3D6 | Down | 135 |
| tgo01230 Biosynthesis of amino acids | Pyruvate kinase | U6KYP2 | Down | 123 |
| tgo01230 Biosynthesis of amino acids | Pyruvate kinase | U6KYP2 | Down | 59 |
| tgo01230 Biosynthesis of amino acids | Pyruvate kinase | U6KYP2 | Down | 210 |
| tgo01230 Biosynthesis of amino acids | Pyruvate kinase | U6KYP2 | Down | 310 |
| tgo01230 Biosynthesis of amino acids | Pyruvate kinase | U6KYP2 | Down | 102 |
| tgo01230 Biosynthesis of amino acids | Pyruvate kinase | U6KYP2 | Down | 211 |
| tgo01230 Biosynthesis of amino acids | Triosephosphate isomerase | H9BA04 | Down | 239 |
| tgo01230 Biosynthesis of amino acids | Triosephosphate isomerase | U6KFY1 | Down | 70 |
| tgo01230 Biosynthesis of amino acids | Fructose-bisphosphate aldolase | U6KYI1 | Down | 38 |
| tgo01230 Biosynthesis of amino acids | Fructose-bisphosphate aldolase | U6KYI1 | Down | 24 |
| tgo01230 Biosynthesis of amino acids | Glyceraldehyde-3-phosphate dehydrogenase | E3VWM6 | Down | 229 |
| tgo01230 Biosynthesis of amino acids | Aconitate hydratase | U6L9U2 | Down | 453 |
| tgo01230 Biosynthesis of amino acids | Aconitate hydratase | U6L9U2 | Down | 893 |
| tgo01230 Biosynthesis of amino acids | Aconitate hydratase | U6L9U2 | Down | 872 |
| tgo01230 Biosynthesis of amino acids | Aconitate hydratase | U6L9U2 | Down | 898 |
| tgo01230 Biosynthesis of amino acids | Transketolase | U6KUY8 | Up | 7 |
| tgo01230 Biosynthesis of amino acids | Transketolase | U6KUY8 | Down | 691 |
| tgo01230 Biosynthesis of amino acids | Phosphoglycerate kinase | U6L4Y7 | Down | 158 |
| tgo01230 Biosynthesis of amino acids | "Isocitrate dehydrogenase, putative (Fragment)" | U6KT00 | Up | 163 |
| tgo01230 Biosynthesis of amino acids | "Isocitrate dehydrogenase, putative (Fragment)" | U6KT00 | Up | 159 |
| tgo01230 Biosynthesis of amino acids | "Isocitrate dehydrogenase, putative (Fragment)" | U6KT00 | Up | 155 |
| tgo01230 Biosynthesis of amino acids | "Gamma-glutamyl phosphate reductase, putative" | U6KLJ2 | Down | 44 |
| tgo01230 Biosynthesis of amino acids | Uncharacterized protein | H9BA08 | Down | 163 |
| tgo01230 Biosynthesis of amino acids | Uncharacterized protein | H9BA08 | Down | 219 |
| tgo01230 Biosynthesis of amino acids | "Aminotransferase, putative" | U6KUP2 | Up | 147 |
| tgo01230 Biosynthesis of amino acids | Enolase | Q967Y8 | Down | 246 |
| tgo01230 Biosynthesis of amino acids | Enolase | Q967Y8 | Down | 131 |
| tgo01230 Biosynthesis of amino acids | Enolase | Q967Y8 | Down | 342 |
| tgo01230 Biosynthesis of amino acids | Enolase | Q967Y8 | Down | 206 |
| tgo01230 Biosynthesis of amino acids | Enolase | Q967Y8 | Down | 264 |
| tgo01230 Biosynthesis of amino acids | Enolase | Q967Y8 | Down | 406 |
| tgo00010 Glycolysis / Gluconeogenesis | Pyrophosphate--fructose 6-phosphate 1-phosphotransferase | U6L3G0 | Down | 667 |
| tgo00010 Glycolysis / Gluconeogenesis | Pyrophosphate--fructose 6-phosphate 1-phosphotransferase | U6L3G0 | Down | 428 |
| tgo00010 Glycolysis / Gluconeogenesis | Pyrophosphate--fructose 6-phosphate 1-phosphotransferase | U6L3G0 | Down | 235 |
| tgo00010 Glycolysis / Gluconeogenesis | Pyrophosphate--fructose 6-phosphate 1-phosphotransferase | U6L3G0 | Down | 195 |
| tgo00010 Glycolysis / Gluconeogenesis | Probable ATP-dependent 6-phosphofructokinase | U6KHR5 | Down | 542 |
| tgo00010 Glycolysis / Gluconeogenesis | Phosphoglycerate mutase | U6L3D6 | Down | 18 |
| tgo00010 Glycolysis / Gluconeogenesis | Phosphoglycerate mutase | U6L3D6 | Down | 61 |
| tgo00010 Glycolysis / Gluconeogenesis | Phosphoglycerate mutase | U6L3D6 | Down | 227 |
| tgo00010 Glycolysis / Gluconeogenesis | Phosphoglycerate mutase | U6L3D6 | Down | 135 |
| tgo00010 Glycolysis / Gluconeogenesis | Pyruvate kinase | U6KYP2 | Down | 123 |
| tgo00010 Glycolysis / Gluconeogenesis | Pyruvate kinase | U6KYP2 | Down | 59 |
| tgo00010 Glycolysis / Gluconeogenesis | Pyruvate kinase | U6KYP2 | Down | 210 |
| tgo00010 Glycolysis / Gluconeogenesis | Pyruvate kinase | U6KYP2 | Down | 310 |
| tgo00010 Glycolysis / Gluconeogenesis | Pyruvate kinase | U6KYP2 | Down | 102 |
| tgo00010 Glycolysis / Gluconeogenesis | Pyruvate kinase | U6KYP2 | Down | 211 |
| tgo00010 Glycolysis / Gluconeogenesis | Triosephosphate isomerase | H9BA04 | Down | 239 |
| tgo00010 Glycolysis / Gluconeogenesis | Triosephosphate isomerase | U6KFY1 | Down | 70 |
| tgo00010 Glycolysis / Gluconeogenesis | Fructose-bisphosphate aldolase | U6KYI1 | Down | 38 |
| tgo00010 Glycolysis / Gluconeogenesis | Fructose-bisphosphate aldolase | U6KYI1 | Down | 24 |
| tgo00010 Glycolysis / Gluconeogenesis | Dihydrolipoyl dehydrogenase | U6KLT6 | Down | 284 |
| tgo00010 Glycolysis / Gluconeogenesis | "Phosphoglucomutase/parafusin related protein 1, putative" | U6KU02 | Down | 155 |
| tgo00010 Glycolysis / Gluconeogenesis | "Phosphoglucomutase/parafusin related protein 1, putative" | U6KU02 | Down | 194 |
| tgo00010 Glycolysis / Gluconeogenesis | Phosphotransferase | A0A172WCE4 | Down | 43 |
| tgo00010 Glycolysis / Gluconeogenesis | Glyceraldehyde-3-phosphate dehydrogenase | E3VWM6 | Down | 229 |
| tgo00010 Glycolysis / Gluconeogenesis | "Alcohol dehydrogenase class III, putative" | U6KWP5 | Up | 234 |
| tgo00010 Glycolysis / Gluconeogenesis | Phosphoglycerate kinase | U6L4Y7 | Down | 158 |
| tgo00010 Glycolysis / Gluconeogenesis | Enolase | Q967Y8 | Down | 246 |
| tgo00010 Glycolysis / Gluconeogenesis | Enolase | Q967Y8 | Down | 131 |
| tgo00010 Glycolysis / Gluconeogenesis | Enolase | Q967Y8 | Down | 342 |
| tgo00010 Glycolysis / Gluconeogenesis | Enolase | Q967Y8 | Down | 206 |
| tgo00010 Glycolysis / Gluconeogenesis | Enolase | Q967Y8 | Down | 264 |
| tgo00010 Glycolysis / Gluconeogenesis | Enolase | Q967Y8 | Down | 406 |

Table S2 Protein domain enrichment analysis of differentially acetylated proteins in sporozoites and merozoites.

| **Domain Description** | **Protein description** | **Protein accession** | **Regulated Type** | **Position** |
| --- | --- | --- | --- | --- |
| Protein kinase domain | cGMP-dependent protein kinase | Q8MMZ8 | Up | 727 |
| Protein kinase domain | "Membrane-attack complex / perforin domain-containing protein, putative" | U6KVP1 | Up | 241 |
| Protein kinase domain | "Rhoptry kinase family protein ROP25, putative" | U6KH66 | Up | 661 |
| Protein kinase domain | "Rhoptry kinase family protein ROP17, putative" | U6KG78 | Up | 285 |
| Aldolase-type TIM barrel | "Ribulose-phosphate 3-epimerase, putative" | U6KT42 | Down | 49 |
| Aldolase-type TIM barrel | Transaldolase | U6L0I8 | Down | 175 |
| Aldolase-type TIM barrel | Triosephosphate isomerase | H9BA04 | Down | 239 |
| Aldolase-type TIM barrel | Triosephosphate isomerase | U6KFY1 | Down | 70 |
| Aldolase-type TIM barrel | Fructose-bisphosphate aldolase | U6KYI1 | Down | 38 |
| Aldolase-type TIM barrel | Fructose-bisphosphate aldolase | U6KYI1 | Down | 24 |
| Aldolase-type TIM barrel | Inosine-5'-monophosphate dehydrogenase | U6KT40 | Down | 457 |
